# Unexpected human cases of cutaneous anthrax in Latium region, Italy, August 2017: integrated human–animal investigation of epidemiological, clinical, microbiological and ecological factors

**DOI:** 10.2807/1560-7917.ES.2019.24.24.1800685

**Published:** 2019-06-13

**Authors:** Emanuele Nicastri, Francesco Vairo, Paola Mencarini, Antonio Battisti, Chiara Agrati, Eleonora Cimini, Stefania Carrara, Silvia D’Arezzo, Rosanna Adone, Antonella Vulcano, Marco Iannetta, Alessandro Capone, Nicola Petrosillo, Antonio Fasanella, Giuseppe Ippolito, Italian Anthrax Team members

**Affiliations:** 1 National Institute for Infectious Diseases Lazzaro Spallanzani – IRCCS, Rome, Italy; 2 These authors contributed equally to this article; 3 Istituto Zooprofilattico Sperimentale del Lazio e della Toscana, Rome, Italy; 4 Istituto Superiore di Sanità, Rome, Italy; 5 Istituto Zooprofilattico Sperimentale Puglia e Basilicata, Foggia, Italy; 6 The members of the network are listed at the end of the article

**Keywords:** *Bacillus anthracis*, cutaneous anthrax, Italy, zoonotic infections, anthrax, outbreaks, surveillance

## Abstract

On 31 August, a veterinarian and a farmworker were hospitalised for skin lesions. Both had been exposed to a dead cow on 19 August on a farm near Rome, where eight further cattle died of confirmed anthrax later the same month. At admission, the first case showed a black depressed eschar and another smaller lesion on one hand. The second case presented deep infection of the skin, with involvement of both arms. Anthrax diagnosis was confirmed by detection of *B. anthracis* DNA in eschar fragments from both patients. T-cell specific immunity was studied by flow cytometry and Elispot assay after stimulation with *B. anthracis* secretome in blood samples collected from Case 1. Immunoglobulin production was detected by complement fixation assay. In Case 1, specific CD4^+^ T-cell activation was detected, without antibody production. Specific antibodies were detected only in the second patient with severe cutaneous illness. Both patients recovered. The two human anthrax cases were epidemiologically linked, but anthrax was not suspected at admission in either case. The veterinarian had initially unrecognised professional exposure and the exposed farmworker did initially not report exposure to affected animals. A One Health strategy integrating human and animal investigations was essential to confirm the diagnosis.

## Introduction

Anthrax is a zoonotic disease and a global health issue [1]. The aetiological agent is *Bacillus anthracis,* a Gram-positive, aerobic, spore-forming and rod-shaped bacterium. Soil is the main reservoir. The disease most commonly affects wild and domestic mammals, mainly herbivores. Humans are secondarily infected by contact with infected animals and contaminated animal products or by direct exposure to *B. anthracis* spores. Each animal dying of anthrax produces enormous quantities of the bacterium in its tissues. If the carcass is opened or when the haemorrhagic secretions or excretions are exposed to the air, the vegetative bacilli convert to resistant spores which contaminate soil, grass and local water sources [1]. Veterinary management of single cases, correct destruction of carcasses, quarantine and vaccination of exposed animals are efficient infection control measures to contain the spread of the disease.

Larger outbreaks of animal anthrax in Italy occurred in Basilicata Region in 2004 and 2011, with 125 and 30 animal infections, respectively [2,3]. In other regions in Italy, animal anthrax is very rare but sporadic outbreaks can occur. Outbreaks were reported in Lazio Region in cattle in 1997 and 2000 and in sheep in 2005 and 2016, involving few animals [4]. In 2016, one confirmed case of ovine infection was reported in Artena, southeast of Rome.

Human disease is now rare in Italy: six cases of human anthrax have been observed since 2004; all were cutaneous forms, affecting veterinary professionals or farmworkers [2,5,6].

On 25 August 2017, the Veterinary Service Area A (Animal Health) of the Azienda Sanitaria Locale Roma 6 (ASL RM6) and the veterinarians of the Regional Veterinary Public Health Institute for Latium and Tuscany (Istituto Zooprofilattico Sperimentale del Lazio e della Toscana, IZSLT) suspected anthrax in four cattle that suddenly died on a pasture in Municipality of Grottaferrata, in the province of Rome, Italy [4,7]. Incomplete rigor mortis and blood oozing from the nostrils were signs consistent with a suspicion of anthrax. At IZSLT, blood smears stained by McFaydean reaction were examined and were positive for *B. anthracis* vegetative forms. On 28 August, all cases were confirmed by culture and PCR (chromosomal target, pXO1 and pXO2 plasmidic targets) [8]. At the end of this outbreak, nine cows of the same herd of 73 died of anthrax [7].

On 31 August, a veterinarian was hospitalised at ‘Lazzaro Spallanzani’ National Institute for Infectious Diseases in Rome for necrotising skin lesions. On the same day, a second patient was admitted in the same hospital for a suspected necrotising fasciitis.

The aim of this report was to describe an exceptional and unexpected outbreak of animal–human anthrax that happened near Rome in 2017.

## Case descriptions

### Case 1

On 19 August 2017, a veterinarian in his 50s inspected a cow that had died of digestive haemorrhage in the same herd in the municipality where anthrax would be identified in several animals in the following week. The veterinarian was accompanied by a groom who helped support the animal's head. Both wore gloves. At that moment, there were no other sick animals and there was no concern about a possible outbreak of anthrax.

The veterinarian had contaminated his left hand with the animal’s blood during removal of the disposable gloves. Ten days later, on 29 August, he noticed the appearance of two skin lesions on his left hand. As he was affected by psoriasis, he considered them psoriatic lesions and applied topical steroids, but 24 h later, the lesion on the index finger evolved to a black eschar, surrounded by erythema and oedema. On 30 August, he self-prescribed one tablet of 500 mg azithromycin and then one tablet of 500 mg ciprofloxacin in the evening and in the morning.

On 30 August, he was alerted by his colleagues at the Veterinary Service that a further four animals had died in the herd he had visited on 19 August and that they had been confirmed with laboratory methods as anthrax cases on 28 August. Only then did he realise that he had probably contracted cutaneous anthrax. On the following day, 31 August, he presented to INMI Spallanzani for consultation.

Considering the clinical diagnosis of cutaneous anthrax, the Regional Epidemiological Service of Surveillance and Control of Infectious Diseases was alerted. The Regional Veterinary Service (IZSLT) confirmed that on 25 August 2017, four bovine animals had died in a pasture in a municipality near Rome, Italy and on 28 August, all cases had been confirmed as anthrax cases [7].

**Figure 1:**
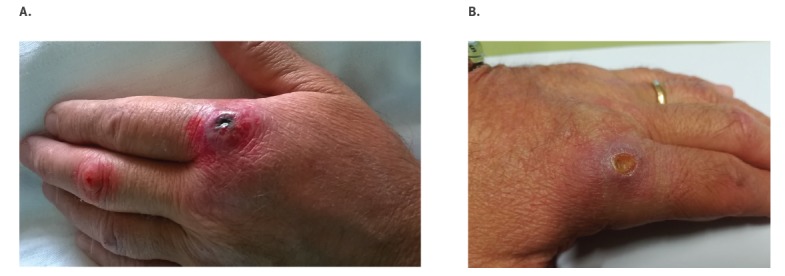
Cutaneous anthrax lesions on (A) Day 13 and (B) Day 36 after exposure, Case 1, Italy, August 2017

The patient was in good clinical condition, without fever. At the base of index finger there was a painless black eschar depressed centrally (1.5 cm diameter), surrounded by a serpiginous blister. A clear halo surrounded the ulcer and oedema was progressing towards the wrist. Intravenous ciprofloxacin (400 mg twice/day) was started and promptly improved the cutaneous lesions (Figure 1, Day 13 from exposure). The patient was discharged after 4 days of hospitalisation with prescription of oral ciprofloxacin (500 mg twice/day for another 15 days). The evolution of the lesions in the following days is documented in Figure 1.

**Figure 2:**
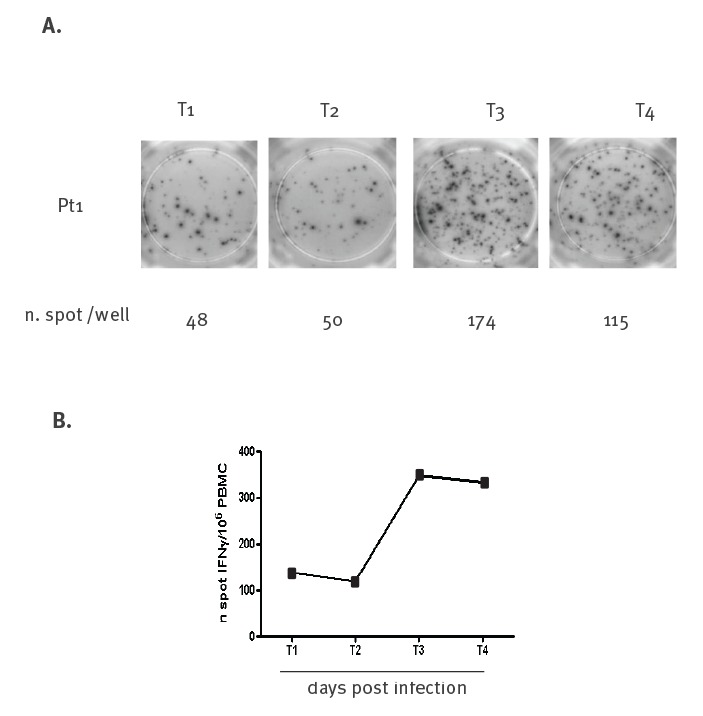
Specific T-cells response in cutaneous anthrax, (A) n. spot /well and (B) n. spot IFNγ/10^6^ PBMC, ELIspot assay, Italy, August 2017

**Figure 3:**
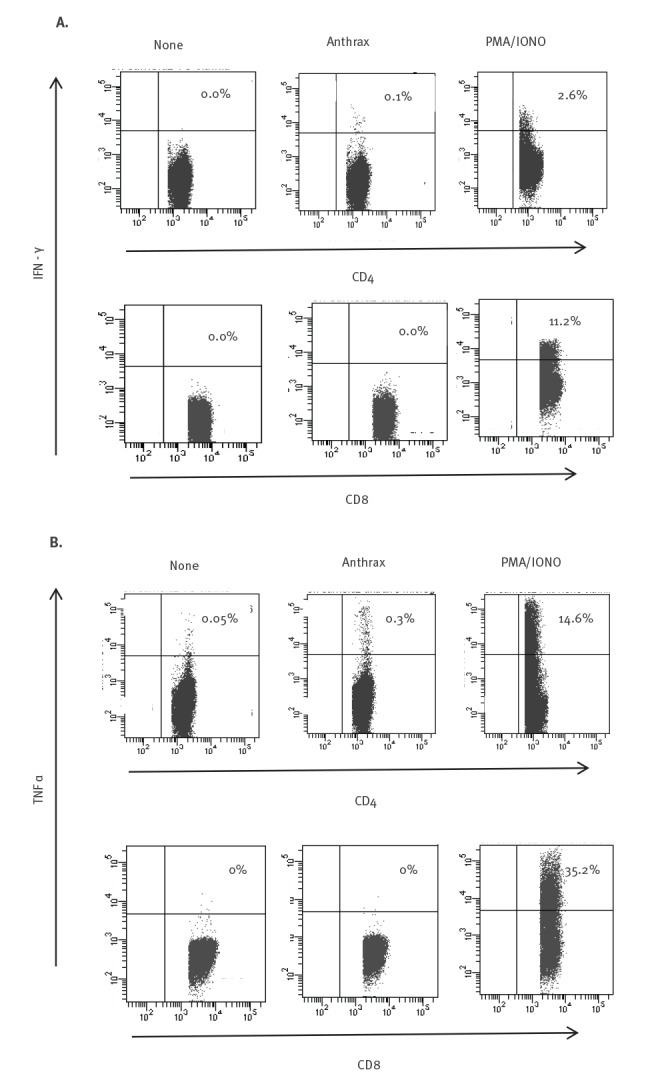
(A) IFNγ and (B) TNFα production in cutaneous anthrax, flow cytometry assay, Italy, August 2017

Culture and PCR of the fluid surrounding the eschar were negative, while the eschar fragment was positive in PCR for *B. anthracis* DNA [9-11]. Specific T-cell response to *B. anthracis* was assessed on peripheral blood mononuclear cells (PBMC) (Figure 2) and flow cytometry (Figure 3). A specific T-cell response was present at Day 12 from exposure and increased with time until Day 24. We observed relevant production of both interferon γ (IFNγ) (Figure 3A) and tumour necrosis factor α (TNFα) (Figure 3B) by the patient’s CD4^+^ T-cells in response to *B. anthracis* stimulation. No specific antibodies to *B. anthracis* were observed during the follow-up on Day 12 and Day 73 post-exposure [12] (see Materials and Methods in the Supplement).

IFNγ: interferon γ; PBMC: peripheral blood mononuclear cells; Pt1: patient 1; SFC: spot-forming cells.

Presence of specific T-cells on Day 12 after exposure (T1: 136 SFC/million PBMC); the response increased over time until Day 24 (T3: 346 SFC/million PBMC) and reached a stable level until Day 73 (T4: 333 SFC/million PBMC).

IFNγ: interferon γ; PMA/IONO: phorbol 12-myristate 13-acetate/ionomycine; TNFα: tumor necrosis factor α.

Ability of CD4^+^ and CD8^+^ T-cell subsets to produce IFNγ (panel A) and TNFα (panel B) in response to *Bacillus anthracis*-specific stimulation. CD4^+^ T-cells represented the main T-cell population involved in the specific response and were able to produce both IFNγ and TNFα.

### Case 2

A man in his 40s looked for medical care at the local Emergency Department in a city 30 km from Rome, on 25 August and 31 August. He presented with several vesicular lesions on his right forearm and was initially treated with topical steroid therapy and parenteral ceftriaxone (1 g/day). Because his clinical condition deteriorated, he was referred to the Spallanzani Institute on 31 August, to a clinical unit different from that of Case 1.

**Figure 4:**
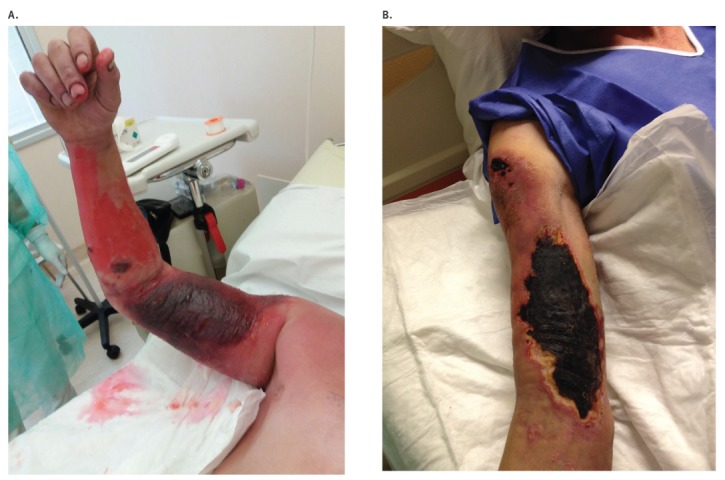
Cutaneous anthrax lesions on (A) Day 13 and (B) Day 20 after exposure, Case 2, Italy, August 2017

At admission, he was in critical condition, with relevant bilateral oedema of the upper extremity up to the shoulders, associated with ulcerated and necrotising skin lesions covered by black eschars (Figure 4). He revealed to be a farmworker but did not report any contact or exposure to sick animals. He had worked on a horse farm bordering the one where the anthrax epidemic had occurred.

Intravenous meropenem (1 g every 3 h), daptomycin (350 mg every 24 h) and clindamycin (600 mg every 6 h) were prescribed and his clinical condition improved promptly in the first 24 h.

Considering the similar clinical presentation of the two cases, the likely professional exposure and the geographical and suspected epidemiological link, a new clinical assessment was performed 24 h later and the patient finally recalled that he had been exposed to the blood of the dead cow inspected on 19 August by Case 1. The previous antibiotic therapy was stopped and he was successfully treated with intravenous ciprofloxacin (400 mg twice/day) and discharged after two weeks after hospitalisation. Oral ciprofloxacin (500 mg twice/day) was prescribed for a further 7 days.

Cultures of swabs from the blisters and ulcers of this patient were all negative, while the PCR for *B. anthracis* DNA from margin eschar fragments taken on 6 September was positive. Specific antibodies to *B. anthracis* were detected with a seroconversion during the convalescent phase at a titre dilution of 1:16 on Day 37 after exposure.

## Control measures

For the control of the outbreak, the Veterinary Service Area A - Animal Health of the Azienda Sanitaria Locale Roma 6 (ASL RM6) made the following provisions: all animals of the affected herd were rapidly moved to a confined area away from the infected area and were kept under sanitary constraint and prohibition of movement. Frequent controls were made to ascertain the occurrence of new cases of disease or death of animals. Vaccination was provided as soon as possible for the entire group of animals on the affected farm and for susceptible animal species (cattle, sheep, goats, pigs, horses) on neighbouring holdings. Daily inspection of the affected herd was performed for an additional 15 days after the affected herd had been vaccinated. No other animal anthrax cases occurred after the animals were vaccinated. The outbreak was declared resolved on 10 October 2017 [13].

## Discussion

This is a report of two epidemiologically linked human anthrax cases in a rural area surrounding the city of Rome: one cutaneous case occurred in a veterinarian of the Latium Regional Public Health System after a professional exposure on 19 August, with a mild clinical presentation. The other case was severe and occurred in a farmworker who initially did not report any exposure to affected animals. At hospitalisation, when he was again questioned about the likely exposure, he confirmed exposure only with delay.

Both cases were eventually attributed to direct exposure to the same animal that died for *B. anthracis* septicaemia. Seven months after these human cases, in March 2018, another cattle was found dead with a microbiologically confirmed anthrax infection on the same pastures [14]. The animal belonged to a herd of unvaccinated cattle that had been translocated to those pastures by mistake, before being vaccinated. In Italy herds of susceptible ruminants are not routinely vaccinated because the disease is rare. Only animals kept in a geographical area where an outbreak has recently occurred are vaccinated in order to protect the susceptible population.

The 2017 cattle outbreak may have been favoured by the severe summer drought in central Italy: cattle at pasture may ingest higher quantities of soil and soil ingestion is thought to be a determinant factor for anthrax infection particularly in dry season [15]. It is noteworthy that the most recent previous outbreak in the pastures of that municipality dates back to 1990, and this may have favoured the loss of concern about the illness among local farmers, practitioners and veterinary services [16].

No specific antibodies to *B. anthracis* were observed in the first mild case. Early antibiotic treatment can prevent the development of a detectable antibody titre [17]. However, molecular biology testing was successful in both human cases, even after initiation of antibiotic treatment.

The natural infection or exposure to *B. anthracis* induces the expansion and differentiation of specific T-cells, with growing evidence that cellular immune responses involving IFNγ-producing CD4^+^ T-cells contribute significantly to protective immunity [18-20]. In Case 1, a *B. anthracis*-specific CD4^+^ T-cell response was observed and maintained over time (at least until Day 73 after infection), even in the absence of a measurable antibody response. Moreover, in accordance with data about the prevalence of a polyfunctional T-cell profile in natural infection [21,22], we observed *B. anthracis* CD4^+^ T-cells able to produce both IFNγ and TNFα [22].

## Conclusion

These two anthrax cases highlight the importance of a concerted One Health response between clinicians and veterinarians, the healthcare delivery system and public health officials. Coordination among veterinary services, local and referral hospitals, epidemiology services and research institutions allowed the identification of two cases of cutaneous human anthrax associated with this outbreak and confirmed the reliability of the One Health approach for surveillance of zoonoses.

Further steps are needed to strengthen the epidemiological, clinical and diagnostic competence about old diseases remerging today. Anthrax should be included in the differential diagnosis of skin lesions; accurate investigation of epidemiological and occupational aspects is needed in selected categories of workers; ecological and environmental contamination of the soil by *B. anthracis* needs to be considered during dry seasons in areas where anthrax has historically occurred.

## Italian Anthrax Team

National Institute for Infectious Diseases ‘Lazzaro Spallanzani’ - IRCCS: Antonino Di Caro, Antonio Mazzarelli, Carla Nisii, Maria Grazia Paglia, Paola Scognamiglio, Carolina Venditti.

Istituto Zooprofilattico Sperimentale del Lazio e della Toscana: Andrea Caprioli, Alessia Franco.

Istituto Zooprofilattico Sperimentale Puglia e Basilicata: Adelia Donatiello, Luigina Serrecchia.

Azienda Sanitaria Locale Roma 6: Mariano Sigismondi, Caterina Aiello.

Lazio Regional Health Service: Domenico Di Lallo, Rita Marcianò.

## Supplementary Data

651000 Supplement S1
