# Supplementary material for: Unexpected human cases of cutaneous anthrax in Latium region, Italy, August 2017: integrated human–animal investigation of epidemiological, clinical, microbiological and ecological factors
Source: Euro Surveill. 2019 Jun 13;24(24):1800685. doi: 10.2807/1560-7917.ES.2019.24.24.1800685 (PMC6582513; doi:10.2807/1560-7917.ES.2019.24.24.1800685)
Supplement: Supplement S1 [file 1800685_Mencarini_SupplementS1.pdf]

Supplement 1: Laboratory Methods for diagnosis of anthrax and for immunological study of the infection in humans

"This supplementary material is hosted by Eurosurveillance as supporting information alongside the article [**Unexpected human cases of cutaneous anthrax in Latium region, Italy, August 2017: integrated human–animal investigation of epidemiological, clinical, microbiological and ecological factors**] on behalf of the authors who remain responsible for the accuracy and appropriateness of the content. The same standards for ethics, copyright, attributions and permissions as for the article apply. Eurosurveillance is not responsible for the maintenance of any links or email addresses provided therein."

- **Cultures and *B. anthracis* identification**: Blood samples of the four cows were cultured in 5% horse sheep blood agar [17]. After overnight incubation at 37°C, typical *B. anthracis* colonies (grey, non-hemolytic, with a ground glass surface) were detected from the samples of all four animals.
- **PCR methods:** suspect isolates of *B. anthracis* were identified according to International Standards, targeting DNA sequences of pOX1 and pOX2 [17], and chromosome specific sequences [18, 19]. DNA was extracted with Qiacube Platform using QIAamp® DNA Mini Kit (QIAGEN) with an additional pre-incubation step with lysozime buffer at 37° for 1 h, proteinase K and AL buffer at 56° for 45 minute. DNA was eluted in 50 ml AE buffer. A *Bacillus* species-specific sequence (*B. anthracis* Pasteur #2H) of chromosomal region (DNA-gyrase subunit b) was also amplified with a PCR assay based on the method of Yamada *et al* [20]. Bacterial primers used to amplify a 245 bp fragment of the gyrB gene were: BA1 – AATCGTAATATTAAACTGACG; and BA2r – CCTTCATACGTGTGAATGTTG. All PCR reactions were performed in a total volume of 50 µl containing 5 µl of template DNA, 0.25 U Taq DNA Polymerase (Invitrogen by Thermo Fisher Scientific.), PCR Buffer (Invitrogen by Thermo Fisher Scientific), 200 mM of each dNTP (SIGMA.), 200 nM of each primer and 1 mM $MgCl_2$. To verify the presence of plasmids, the gene encoding the edemigenous factor was identified: plasmid marker pXO1 and the coding gene for unit A of the capsular protein complex CAP: pXO2 plasmid marker [18].
- ***Flow cytometry :*** T-cell specific immunity was evaluated by flow cytometry at different time points: at 12 days (T1), 18 days (T2), 24 days (T3) and 73 days (T4) from exposure. Briefly, Peripheral Blood Mononuclear Cells (PBMC) were isolated by Ficoll procedure, counted and stored at −80. Cryopreserved PBMC were rapidly thawed, washed with culture medium (RPMI 1640, 10% FCS, 2 mmM L-glutamine, penicillin 50 IU/ml, streptomycin 50 µg/ml), cultured $1x10^6$ cells/ml, stimulated with *B. anthracis* secretome at 5µg/ml and PMA/Ionomycin (PMA 50 nM and Ionomycin 1µM, Sigma Aldrich) for 24 hours. Brefeldin A (10 µg/ml, Serva) was added after one hour of stimulation. Cells were stained and analyzed by flow cytometry using the following anti-human monoclonal antibodies to assess T-cell subsets and T-cell cytokines production: CD4 V450, CD8 PeCy7, CD3 PerCp Cy 5.5, TNF-α FITC, IFN-γ-PE (BD Pharmigen). Briefly, PBMC were incubated for 20 min at 4 °C with anti-CD3, anti-CD4 and anti-CD8 mAbs, washed with buffer (PBS/1%BSA/0.1% sodium azide), and fixed with 1% paraformaldehyde 5 minutes at RT. After washing, cells were stained with anti-TNFα/anti-IFNγ in buffer (PBS/1%BSA/0.1% sodium azide, 0.5% Saponin) 20 minutes at RT, washed and acquired using

a FACSCanto II flow cytometer (Becton Dickinson). Data analysis were performed with DIVA software (Becton Dickinson).

- ***Elispot assay:*** T-cell functionality during *B. anthracis* infection was assessed by detecting interferon-gamma (IFNγ) production using an enzyme-linked immunosorbent spot-forming cell assay (ELISpot) after stimulation. PBMCs were thawed in culture medium (RPMI 1640, 10% FCS, 2 mmM L-glutamine, penicillin 50 IU/ml, streptomycin 50 µg/ml) and assessed for vitality by Trypan Blue exclusion, counted, and plated at 3×10^5 cells/well in ELISpot plates (AID GmbH, Strabberg, Germany). PBMCs were then stimulated with *B. anthracis* secretome at 5 µg/ml and PHA, included in the Elispot kit, for 24 hours with 5% of CO_2. At the end of incubation, the ELISpot assay was developed according to manufacturer's instructions. Spontaneous cytokine production (background) was assessed by incubating PBMC with 1 µg/ml αCD28 and αCD49d (IgG1, clones CD28.2 and 9f10, respectively; Becton Dickinson). Results are expressed as spot forming cells (SFC)/10^6 PBMCs in stimulating cultures after subtracting spontaneous background.
- **Complement fixation test**: serum samples of the two patients were tested with the Sterne-based CFT which utilizes an inactivated suspension of *B. anthracis* strain $34F_2$ as antigen, as previously described by Adone et al., 2016 [21]. Sera were diluted 1:2 in Veronal buffer and incubated for 30 min at 56°C to inactivate the native complement. Then, in 96-well round-bottom plates 25 µl of each serum was serially diluted from 1:2 to 1:128 prior to adding 25 µl of antigen and 25 µl of complement at working dilution. Plates were incubated at 37°C for 30 min and then 25 µl of sensitized erythrocytes were added to each well. After incubation, plates were centrifuged at 2000 g for 5 min to allow any unlysated cells to deposit and the reaction was read over a diffused white light: 100% hemolysis was considered as negative reaction, while all reactions showing complete absence of hemolysis (0%) or partial hemolysis 75, 50, or 25% were considered as positive. The titer of each serum was the highest dilution showing a positive reaction; the serum dilution 1:2 showing 50% of hemolysis was taken as the reactivity threshold of the reaction. A bovine, hyper immune serum containing anti-anthrax antibodies was used as positive control and a pool of human sera from healthy people as negative control.
